# Changes in testosterone related to body composition in late midlife: Findings from the 1946 British birth cohort study

**DOI:** 10.1002/oby.21092

**Published:** 2015-06-05

**Authors:** David Bann, Frederick C. W. Wu, Brian Keevil, Hany Lashen, Judith Adams, Rebecca Hardy, Graciela Muniz, Diana Kuh, Yoav Ben‐Shlomo, Ken K. Ong

**Affiliations:** ^1^ MRC Unit for Lifelong Health and Ageing at UCLLondonUK; ^2^ Andrology Research Unit, School of BiomedicineUniversity of ManchesterManchesterUK; ^3^ Department of Human MetabolismThe University of SheffieldSheffieldUK; ^4^ Department of RadiologyCentral Manchester University Hospital NHS Foundation Trust, Manchester Academic Health Science CenterManchesterUK; ^5^ School of Social and Community MedicineBristol UniversityBristolUK; ^6^ MRC Epidemiology UnitUniversity of CambridgeCambridgeUK.

## Abstract

**Objective:**

Randomized trials in men with testosterone deficiency have provided evidence of short‐term effects of testosterone therapy on muscle and fat mass but it is unclear whether this persists over a longer period or how testosterone affects women. We examined whether the midlife decline in testosterone relates to fat and lean mass in both sexes.

**Methods:**

Data were collected from 440 men and 560 women participating in the 1946 British birth cohort study with testosterone measured at 53 and/or 60‐64 years. Fat and appendicular lean mass were measured at 60‐64 years using dual‐energy X‐ray absorptiometry.

**Results:**

Mean free testosterone concentrations were lower at 60‐64 than 53 years, by 26% in both sexes. At both ages testosterone was negatively associated with fat mass in men and positively associated in women. A larger decline in free testosterone was associated with higher fat mass in men but with lower fat mass among women. In contrast, declines in testosterone were not associated with lean mass in either sex.

**Conclusions:**

Our findings suggest sex‐divergent relationships between testosterone and fat mass and their distribution but do not support the hypothesis that midlife declines in testosterone lead to lower lean mass.

## Introduction

Testosterone is known to have a number of important regulatory roles in adults, and its decline observed during aging 1, 2 may adversely affect physical health and functioning via gains in fat and losses in lean (muscle) mass 3, 4. An evidence‐based understanding of the changes in testosterone that occur with aging—and the consequences of these changes—is important, given recent trends in testosterone prescribing; among men in the UK and USA, there has been an almost twofold and threefold increase in testosterone prescription from 2001 to 2011, respectively 5, 6.

Experimental studies have shown that testosterone supplementation in men with low testosterone concentrations leads to gains in muscle 7 and losses in fat mass 8, 9. Conversely, diet‐ or bariatric surgery‐induced weight loss reverses the pathological suppression of testosterone levels in overweight/obese men 10, suggesting a bi‐directional relationship between testosterone and fat mass. In contrast to these effects in men, rare reports of women undergoing sex change therapy suggest that testosterone supplementation may increase visceral fat mass 11. However, randomized trial evidence is limited to interventions over 1‐3 years in a small number of men with testosterone deficiency. Thus, important questions remain as to the longer term influence of testosterone for aging men and women 12. Observational studies offer the advantage of studying differences in body composition in large numbers of subjects with wide variations in testosterone levels, within individuals over long intervals. However, previous observational studies have mostly been cross‐sectional, usually only of men, and yielded inconsistent results 13.

To the authors' knowledge, this is the first study to prospectively examine the associations between testosterone over a 10‐year period with fat mass, fat distribution, and lean mass in late midlife simultaneously in both men and women. We hypothesized that testosterone has a sexually dimorphic effect on fat but a monomorphic effect on lean mass, so that decline in testosterone would be associated with higher fat mass in men but lower fat mass in women and lower lean mass in both sexes.

## Methods

### Study sample

The MRC National Survey of Health and Development (NSHD) is a socially stratified sample of 5,362 singleton births during 1 week of March 1946 in mainland Britain 14, with regular follow‐up across life. Between 2006 and 2010 (at 60‐64 years), 2,856 eligible study members (those known to be alive and with a known address in mainland Britain) were invited for an assessment at one of six clinical research facilities (CRFs) or to be visited by a research nurse at home. Invitations were not sent to those who had died (*n* = 778), who were living abroad (*n* = 570), who had previously withdrawn from the study (*n* = 594), or who had been lost to follow‐up (*n* = 564). Of those invited, 2,229 were assessed: 1,690 attended a CRF and the remaining 539 were seen at home 15. The study received multicenter research ethics committee approval, and informed consent was given by participants.

### Body composition measurement

During CRF visits, body composition measures were obtained in the supine position using a QDR 4500 Discovery dual‐energy X‐ray absorptiometry (DXA) scanner (Hologic Inc, Bedford, MA, USA) with APEX 3.1 software. Measures used as outcomes were whole body fat mass (kg), a measure of fat distribution (android:gynoid ratio (multiplied by 100); higher values indicating greater fat in the abdominal (android) region relative to the hips (gynoid)), and appendicular (limb) lean mass (kg)—1,558 participants had data available for height and body composition outcomes, with missing data largely due to high‐density artifacts.

### Blood sampling and testosterone measurement

At 53 years, nonfasting venous blood samples were obtained during home visits from 2,750 participants in ethylenediaminetetraacetic acid (EDTA) tubes in the morning (27%), afternoon (30%), or evening (43%). These were then posted overnight to a laboratory where plasma was extracted before being frozen at −80°C. At 60‐64 years, overnight fasting venous blood samples were obtained in citrate tubes from 2,143 participants, in most cases in the morning (93% between 7.30 am and 10 am)—these were then taken immediately to a laboratory during CRF visits, and plasma extracted before being frozen at −80°C.

Total testosterone and sex‐hormone binding globulin (SHBG) were measured in the same laboratory from plasma samples at 53 and 60‐64 years. Liquid chromatography‐mass spectrometry (LC‐MS/MS) was used to measure testosterone 16, 17, and immunoassay used for SHBG (Abbott Diagnostics, Maidenhead, UK). Inter‐assay coefficients of variation were as follows: 4.7, 3.9, 4.3, 4.0, and 3.6% for testosterone (at 0.9, 1.7, 4.3, 16.8, and 29.0 nmol/L), and 2.7, 5.0, and 5.4% for SHBG (at 8.4, 22.4, and 136 nmol/L). A total of 1,000 participants had valid testosterone measures at both ages—missing data were largely due to insufficient plasma following other biochemical assays.

### Analytical strategy

As taller individuals tend to have more fat and lean mass, height‐adjusted indices for fat and appendicular lean mass were created by dividing fat and lean mass (kg) by height(*m*)*^X^* (*X* = 1.2 for fat and 2 for lean mass), where *X* was calculated so that the resulting index was not correlated with height 18, 19.

To preserve the analytic sample, values which fell below detection limits (<0.3 nmol/L for testosterone) were replaced with a random value between 0 and the detection limit (total *N* (irrespective of outcome data) = 146 for testosterone (98% women). Values above detection limits were truncated to the detection limit (250 nmol/L for SHBG (*N* = 5)). To minimize the impact of outliers, six women with high testosterone concentrations were truncated at 3 nmol/L—the inclusion of original outlying values was found to bias regression coefficients. Free testosterone was calculated as described by Vermeulen et al. 20—an albumin constant (4.3g/L) was assumed for all participants at 53 years, and measured albumin was used at 60‐64 years.

Linear regression was used to examine associations between hormones and body composition outcomes, separately for each sex. To aid the comparability of effect sizes, hormone measures were converted to sex‐specific *z*‐scores; measures at 53 and 60‐64 years from women were log‐transformed before this as distributions were positively skewed. Outcomes were log transformed and multiplied by 100—regression coefficients therefore show the mean percentage difference in outcome per 1 standard deviation increase in testosterone. We tested for potential sex differences in associations by including an interaction term. We also assessed models with testosterone categorized in quintiles to examine for any departures from linearity.

Samples at 53 years we taken either in the morning, afternoon or evening, unlike morning blood samples at 60‐64 years. We took into account diurnal variability in testosterone (higher concentrations in the morning) by running sex‐specific regression models with testosterone at 53 years as the outcome and time of blood collection as a categorical exposure. The residuals from this model were then added to mean morning testosterone concentration for those who gave blood samples in the afternoon and evening.

We firstly tested associations between testosterone and our outcomes cross‐sectionally (60‐64 years) and prospectively (53 years). We then ran conditional change models by including the change in hormone from 53 to 60‐64 adjusting for hormone concentration at 53 years. Complete case analyses were used in all regression analyses; of the 1,000 participants with valid testosterone measures at both ages, 200 had missing body composition outcome data because they did not attend the CRF at 60‐64 years, and 19 had missing data for potential confounders, leaving an analytic sample of 781.

### Potential confounders

Associations with lean mass could be confounded by fat mass, as due to adaptive mechanisms changes in fat mass typically lead to respective changes in lean mass 21. We therefore examined lean mass without and with adjustment for fat mass index. Models analyzing change in testosterone were further adjusted for additional potential confounders selected a‐priori: highest household occupational class, smoking status, self‐rated health (reported number of health problems from 0‐5) and menopausal status at 53 years (categorized as pre‐, peri‐ or post‐menopausal (5.4, 14.3, and 28.7% of the total sample respectively), hysterectomy (23.4%) or other operation stopping periods (6.0%), and a further 22.1% could not be classified because they started taking HRT before the menopause, as described elsewhere 22.

### Supplementary analyses

To examine the possibility of reverse or bi‐directionality, change models for fat mass were repeated with additional adjustment for body mass index (BMI) at age 53, and further analyses conducted in which BMI (or change in BMI) was the exposure, and testosterone the outcome. To examine the extent to which adjustment for time of blood sampling affected findings, analyses were repeated with no adjustment for time of blood sample; analyses were also repeated restricted to those with morning blood samples at both 53 and 60‐64 years (before 10am at 60‐64 years; total *N* = 219). To examine whether truncation of testosterone among the six participants with outlying values impacted on findings, analyses were repeated excluding these participants. To examine the extent to which analyses of change were non‐linear, analyses were repeated using change score variables converted into quartiles.

## Results

### Descriptive analyses

Men had greater android fat mass, android:gynoid ratio and appendicular lean mass, whilst women had a larger fat mass index and gynoid fat mass (*P* < 0.02 in all cases). As expected, mean total and free testosterone were higher in men than women, while women had higher SHBG concentrations (*P* < 0.001 in all cases). In both sexes, mean testosterone concentrations declined from 53 to 60‐64 years (by approximately 22% among men, and 35% among women). Mean free testosterone also declined (by approximately 26% among men, and 26% among women), while SHBG slightly increased in men and declined in women. There were moderate correlations between total and free testosterone at 53 and 60‐64 suggesting some tracking of hormone concentrations (*r* = 0.66 and *r* = 0.40 in men, *r* = 0.44 and *r* = 0.49 in women, respectively; *P* < 0.001 in all cases). Those with missing testosterone data at 60‐64 years differed in some respects in their hormone concentrations at 53 years—on average men with missing data at 60‐64 years had higher total testosterone (*P* = 0.03) and SHBG (*P* = 0.01) concentrations at 53 years, but there were no significant differences in free testosterone among either sex. At 53 years, women who were post‐menopausal had lower total testosterone (*P*(trend) = 0.02) and lower SHBG (*P*(trend) = 0.01) than pre or peri‐menopausal women but there was no difference in free testosterone (*P*(trend) = 0.58).

### Testosterone and fat mass

Among men, higher total and free testosterone concentrations at both 53 and 60‐64 years were associated with lower fat mass at 60‐64 years (Table 2; categorical analyses shown in Supporting Information Table S1). Unlike men, women with higher testosterone at 53 years and 60‐64 years had higher fat mass, though in the latter confidence intervals overlapped with the null (Table 2 and Supporting Information Table S1; sex interaction tests all *P* < 0.05). Higher SHBG at both ages was associated with lower fat mass in both sexes—more strongly in women than men. Associations between these exposures with android:gynoid ratio and BMI at 60‐64 years (Supporting Information Table S2) were generally similar to those with fat mass.

**Table 1:** Summary of body composition and testosterone concentrations by sex

	Men	Women
	Mean (SD or IQR)	Mean (SD)
***Hormone concentration, age***	*N = 440*	*N = 560*
**Testosterone (nmol/L), 53**	16.2 (4.9)	0.9 (0.4)
**Testosterone (nmol/L), 60–64**	12.6 (4.7)	0.6 (0.3)
**Δ Testosterone**	−3.6 (−6.0, 1.3)^a^	−0.3 (−0.5, −0.1)^a^
**Sex‐hormone binding globulin (nmol/L), 53**	34.2 (14.0)	65.0 (40.6)
**Sex‐hormone binding globulin (nmol/L), 60–64**	36.4 (14.5)	49.4 (26.2)
**Δ Sex‐hormone binding globulin**	2.2 (−3.9, 7.8)^a^	−15.6 (−26.3, 4.4)^a^
**Calculated free testosterone (pmol/L), 53**	325.0 (81.8)	11.9 (7.9)
**Calculated free testosterone (pmol/L), 60–64**	240.1 (68.8)	8.8 (6.0)
**Δ Calculated free testosterone**	−85.0 (−141.0, −34.1)^a^	−3.1 (−5.5, 0.1)^a^
***Body composition at*** ***60*–*64*** ***years***	*N = 746*	*N = 812*
**Fat mass index (kg/m^1.2^)**	12.0 (3.6)	16.2 (5.0)
**Android fat mass (kg)**	2.5 (1.0)	2.3 (1.0)
**Gynoid fat mass (kg)**	3.7 (1.0)	5.1 (1.4)
**Android:gynoid ratio**	65.16 (15.5)	44.9 (12.0)
**Appendicular lean mass index (kg/m^2^)**	8.0 (1.0)	6.2 (0.9)
**Body mass index**	27.7 (3.9)	27.5 (5.0)
***Body mass index at 53 years***	*N = 989*	*N = 1110*
	27.2 (3.8)	27.1 (5.1)

a *P* < 0.001, comparison between ages using paired t‐tests; free testosterone calculated according to Vermeulen et al.^20^; estimated morning testosterone concentrations used at 53 years; analyses restricted to participants with valid data for all body composition outcomes or all hormone measures at both ages. Body mass index at 53 years calculating using the sample with valid body mass index data for 60–64 years.

**Table 2:** Mean percentage differences in fat mass and android:gynoid fat mass ratio (95% CI) at 60–64 years per 1 standard deviation increase in serum testosterone concentration at 53 and 60–64 years

		Men (N = 345)	*P*	Women (N = 436)	*P*	*P* sex interaction
***Fat mass index***						
**Testosterone, 53**		−6.75 (−10.17, −3.32)	<0.001	4.57 (1.65, 7.49)	<0.01	<0.001
**Testosterone, 60–64**		−10.77 (−13.78, −7.75)	<0.001	2.31 (−0.45, 5.07)	0.10	<0.001
**Δ Testosterone**		−9.96 (−13.35, −6.56)	<0.001	0.47 (−3.10, 4.04)	0.80	<0.01
**Δ Testosterone, adjusted** a		−9.54 (−13.03, −6.06)	<0.001	1.19 (−2.43, 4.80)	0.52	<0.01
**SHBG, 53**		−4.59 (−8.17, −1.00)	0.01	−9.11 (−11.96, −6.26)	<0.001	0.04
**SHBG, 60–64**		−5.43 (−8.58, −2.28)	<0.001	−13.39 (−16.14, −10.64)	<0.001	<0.001
**Δ SHBG**		−3.85 (−7.21, −0.49)	0.02	−13.21 (−16.93, −9.50)	<0.001	<0.01
**Δ SHBG, adjusted** a		−3.20 (−6.66, 0.26)	0.07	−11.67 (−15.53, −7.82)	<0.001	<0.01
**Free testosterone, 53**		−5.43 (−8.58, −2.28)	<0.001	9.51 (6.77, 12.25)	<0.001	<0.001
**Free testosterone, 60–64**		−5.12 (−8.39, −1.86)	<0.01	8.39 (5.74, 11.03)	<0.001	<0.001
**Δ Free testosterone**		−11.54 (−15.58, −7.50)	<0.001	3.63 (0.40, 6.87)	0.03	<0.001
**Δ Free testosterone, adjusted** a		−11.31 (−15.42, −7.21)	<0.001	3.98 (0.80, 7.15)	0.01	<0.001
***Android:gynoid fat mass ratio***						
**Testosterone, 53**		−3.42 (−6.19, −0.66)	0.02	3.29 (0.63, 5.95)	0.02	0.003
**Testosterone, 60–64**		−6.67 (−9.14, −4.19)	<0.001	1.66 (−0.85, 4.17)	0.19	<0.001
**Δ Testosterone**		−6.84 (−9.63, −4.06)	<0.001	0.72 (−2.54, 3.98)	0.67	<0.01
**Δ Testosterone, adjusted** a		−6.41 (−9.24, −3.57)	<0.001	1.79 (−1.57, 5.14)	0.30	<0.01
**SHBG, 53**		−4.55 (−7.40, −1.70)	<0.01	−8.86 (−11.43, −6.28)	<0.001	0.88
**SHBG, 60–64**		−5.87 (−8.35, −3.39)	<0.001	−13.46 (−15.90, −11.02)	<0.001	0.34
**Δ SHBG**		−4.57 (−7.21, −1.93)	<0.001	−13.76 (−17.05, −10.47)	<0.001	<0.01
**Δ SHBG, adjusted** a		−4.10 (−6.81, −1.39)	<0.01	−11.90 (−15.39, −8.41)	<0.001	<0.01
**Free testosterone, 53**		−0.36 (−3.00, 2.28)	0.79	8.36 (5.86, 10.86)	<0.001	0.01
**Free testosterone, 60–64**		−4.06 (−6.66, −1.47)	<0.01	6.95 (4.53, 9.37)	<0.001	<0.001
**Δ Free testosterone**		−5.51 (−8.88, −2.15)	<0.01	4.20 (1.26, 7.14)	<0.01	<0.001
**Δ Free testosterone, adjusted** a		−5.07 (−8.47, −1.66)	<0.01	4.79 (1.86, 7.73)	<0.01	<0.001

Δ standard deviation change between 53 and 60–64 years—analyses adjusted for hormone concentration at 53 years (e.g. Δ testosterone adjusted for testosterone at 53 years).

a Adjusted for highest household occupational class, smoking, self‐rated health, and menopausal status at 53 years. SHBG: sex‐hormone binding globulin; free testosterone calculated according to Vermeulen et al.; estimated morning testosterone concentrations used at 53 years.

As predicted, greater declines in total and free testosterone between 53 and 60‐64 years were associated with higher fat mass and higher android:gynoid ratio among men (see negative coefficients in Table 2). In contrast, greater declines in free testosterone were associated with lower fat mass and android:gynoid ratio among women, while total testosterone was not associated. The magnitude of these associations was large for men. In adjusted models, a 1 standard deviation greater decline in free testosterone was associated with 11% higher fat mass in men and 4% lower fat mass in women. In both sexes, greater declines in SHBG (or, equivalently, lesser gains in SHBG) were associated with higher fat mass and android:gynoid ratio. Associations were similar after adjustment for potential confounders, and for BMI at 53 years (Supporting Information Table S3).

### Testosterone and appendicular lean mass

Neither total nor free testosterone at 53 or 60‐64 years were associated with appendicular lean mass at 60‐64 years in men (Table 3). Among women, higher free testosterone at both ages was associated with higher lean mass. In both sexes higher SHBG (at both ages) was associated with lower lean mass.

**Table 3:** Mean percentage differences in appendicular lean mass (95% CI) at 60–64 years per 1 standard deviation increase in serum testosterone concentration at 53 and 60–64 years

	Men (N = 345)	*P*	Women (N = 436)	*P*	*P* sex interaction
**Testosterone, 53**	−0.39 (−1.57, 0.78)	0.51	−0.18 (−1.20, 0.84)	0.73	0.50
**Testosterone, 60–64**	−0.40 (−1.53, 0.74)	0.49	0.27 (−0.68, 1.22)	0.58	0.19
**Δ Testosterone**	−0.22 (−1.48, 1.03)	0.73	0.43 (−0.81, 1.67)	0.49	0.50
**Δ Testosterone, adjusted** a	−0.39 (−1.66, 0.89)	0.55	0.57 (−0.71, 1.85)	0.38	0.32
**SHBG, 53**	−1.42 (−2.62, −0.23)	0.02	−1.81 (−2.86, −0.76)	<0.001	0.82
**SHBG, 60–64**	−1.60 (−2.66, −0.55)	<0.01	−2.53 (−3.63, −1.44)	<0.001	0.93
**Δ SHBG**	−1.06 (−2.18, 0.06)	0.06	−2.06 (−3.48, −0.63)	<0.01	0.05
**Δ SHBG, adjusted** a	−1.24 (−2.39, −0.10)	0.03	−1.76 (−3.25, −0.26)	0.02	0.17
**Free testosterone, 53**	0.45 (−0.66, 1.56)	0.42	0.97 (−0.06, 2.00)	0.07	0.27
**Free testosterone, 60–64**	0.74 (−0.42, 1.89)	0.21	1.16 (0.18, 2.13)	0.02	0.34
**Δ Free testosterone**	0.78 (−0.70, 2.27)	0.30	0.62 (−0.55, 1.79)	0.30	0.51
**Δ Free testosterone, adjusted** a	0.74 (−0.76, 2.25)	0.33	0.71 (−0.49, 1.91)	0.24	0.38

All models are adjusted for fat‐free mass index; Δ standard deviation change between 53 and 60–64 years—analyses adjusted for hormone concentration at 53 years (e.g., Δ testosterone adjusted for testosterone at 53 years).

a Adjusted for highest household occupational class, smoking, self‐rated health, and menopausal status at 53 years. SHBG: sex‐hormone binding globulin; free testosterone calculated according to Vermeulen et al.; estimated morning testosterone concentrations used at 53 years.

Contrary to our predictions, there was little evidence that changes in total or free testosterone were associated with lean mass in either sex either before or after adjustment for fat mass (Table 3). However, greater declines in SHBG were associated with were associated with higher lean mass, both before and after adjustment for potential confounders.

### Additional and sensitivity analyses

Greater gains in BMI from 53 to 60‐64 years were associated with lower testosterone concentrations at 60‐64 years among men (but not women), and greater gains in BMI were weakly (nonsignificantly) associated with lower free testosterone in men, and higher free testosterone among women (Supporting Information Table S4). Findings were similar when not adjusting for time of blood collection at 53 years, when restricting analyses to participants with morning blood samples, or when excluding participants with outlying testosterone values. Finally, there was little evidence for nonlinearity in the change models.

## Discussion

In this representative British birth cohort study, marked declines in total and free testosterone were observed during late midlife (53 to 60‐64 years) in both men and women. Larger declines in free testosterone were associated with higher fat mass among men, yet lower fat mass among women at 60‐64 years. There was little evidence linking declines in testosterone with lean mass in either men or women.

Our data add significantly to previous evidence linking testosterone to body composition outcomes—existing observational studies have mostly been cross‐sectional, conducted in men, and yielded equivocal findings 13, 23, 24. The NSHD enabled the investigation of variations in testosterone and SHBG across the entire male and female population distribution and their relationships to DXA body composition measures. The repeat measures obtained also enabled the longitudinal investigation of how age‐related declines in these hormones related to body composition outcomes.

Sex differences in the relationships between free testosterone and body fat mass may reflect sex differences in the physiological role of testosterone, and/or differences in upstream factors that drive testosterone concentrations. Among men with low testosterone concentrations, supplementation lowers fat mass levels 7, 8, while rare reports of women undergoing sex change therapy indicate that supplementation increases visceral but lowers subcutaneous at 11. In addition, polycystic ovary syndrome is typically characterized by high testosterone concentrations and is associated with higher fat mass. Little is currently known about the physiological roles of testosterone among women. There is emerging evidence (albeit from cross‐sectional studies) that higher endogenous testosterone is related to adverse cardiometabolic health in women 25, despite beneficial relationships with other outcomes such as bone mineral density 24. Sex differences could also be driven by the downstream products of testosterone such as estrogen—in postmenopausal women, circulating estrogen is mostly derived from peripheral conversion of androgens, and fat cells are known to contain estrogen receptors. However, hormone replacement therapy in postmenopausal women reduces abdominal fat mass 26, suggesting that the impacts of estrogen are unlikely to explain the positive associations found between testosterone and fat mass. Further research is clearly needed to better understand the sex differences in relations between testosterone, estrogen and fat mass.

Associations between testosterone and fat mass were similar when adjusting for an earlier indicator of fat mass (BMI at 53 years), suggesting that reverse causality is unlikely to fully explain these findings. However, associations between testosterone and fat mass are likely to be bi‐directional—weight loss have been found to lead to higher testosterone in men 10, yet lower testosterone in women 27. In support of this direction of association, as also found in in other studies 28, 29. Greater gains in BMI across midlife were associated with lower testosterone concentrations among men. Disentangling which direction of association predominates using observational data is likely to be challenging. One previous study compared the magnitude of the testosterone‐body weight association in women with that of the reverse direction (body weight‐testosterone) to infer which direction of association was most likely 30. However, this inference may not be appropriate as regression dilution would result in a weaker magnitude of association between exposure and outcome when the exposure is measured less precisely (i.e., for testosterone (assessed using immuno‐assay) rather than weight).

We found little evidence linking declines in testosterone with lean mass, in contrast to evidence from experimental studies demonstrating anabolic effects of testosterone (7‐9). A possible explanation for this difference is that the current study analysed variations in testosterone across the physiological range, whereas the effects of testosterone administration on lean mass are typically performed in older men with hypogonadal testosterone levels. In addition, testosterone may be more strongly associated with the active tissue components of lean mass (contractile muscle fibers), which DXA does not distinguish from other components such as intra and extra‐cellular fluid 31. Most trials assess short‐term benefits usually for no more than 1 year 32, while our results over a decade may be influenced by other factors that attenuate these short‐term effects. Lower testosterone has been found to be related to greater age‐related decline change in lean mass (among men that lost weight) 33, suggesting that testosterone may be especially important for the processes which underlie this change—this warrants future investigation in cohorts with repeat data. The null findings presented here do not preclude the importance of testosterone for muscle development during early‐life growth.

SHBG was inversely associated with fat mass and android:gynoid ratio in both men and women. This could suggest that SHBG has a role in regulating fat mass levels. In support of this, SHBG genetic variations conferring higher SHBG concentrations have been associated with lower risk of type‐2 diabetes 34, which is thought to be partly determined by fat mass levels. Much like associations between testosterone and fat mass, associations with SHBG are likely to be bi‐directional, as higher fat mass levels would be associated with insulin resistance and therefore higher insulin concentrations, which in turn down‐regulates SHBG 35. This is demonstrated in intervention studies which have found weight loss results in higher SHBG 36. SHBG was also inversely associated with lean mass—future studies should consider whether SHBG has any plausible physiological role in muscle mass regulation, or may be a useful biomarker to indicate muscle mass levels.

Strengths of this study include the repeat measures of testosterone ascertained in late midlife, a period during which the expected decline with age was observed. Despite blood samples being collected across mainland Britain, standard protocols were used and testosterone assays at both ages were assessed in a single laboratory using LC‐MS/MS, which provides accurate testosterone measures even at low concentrations 16, 17. Other strengths include the sampling of both sexes, precise and detailed measures of regional and whole fat and lean mass, and availability of data for potential confounders.

Limitations of this study include the lack of repeat DXA measures of fat and lean mass, which precluded the investigation of how testosterone relates to change in these masses; however, our sensitivity analyses using repeat BMI measures suggest that the findings are likely to be similar. As in all longitudinal studies, attrition occurred which limited statistical power and may have introduced bias (if the associations investigated differed in the group lost to follow‐up). Blood samples were only taken once at each age and although single measures of testosterone have been found to accurately reflect long‐term concentrations 37, multiple measures at more than two ages may better characterize change in testosterone with age. Finally, methodological differences likely affected the estimation of decline in testosterone: at 53 years, nonfasting samples were used 38, and samples were frozen for longer periods, with a longer delay before being frozen—these likely resulted in an underestimation of testosterone at this age, and therefore its decline. Finally, blood samples were taken throughout the day at 53 years but primarily during the morning at 60‐64 years. However, blood sample timing was accounted for in analyses, and similar results were also found when restricting analyses to participants with morning blood samples at both ages.

While the findings from this study suggest that higher testosterone concentrations may have favorable effects on body composition in older men, these do not directly support the pharmacological raising of testosterone levels which may have adverse consequences for cardiovascular health. While one meta‐analysis found testosterone supplementation did not relate to cardiovascular risk 39, a subsequent meta‐analysis found that oral (but not injection or transdermal) administration was related to increased cardiovascular risk 40. Ongoing trials 32 which will compare (in men with low testosterone) the relative benefits of testosterone supplementation for a number of health‐related outcomes. Alternative strategies that focus on up‐stream determinants of testosterone (such as early life or behavior) may potentially lessen age‐related decline and in turn could limit the accumulation of fat mass and losses in lean mass that occur during aging. Further research is clearly needed to identify the modifiable determinants of testosterone and its decline with age.

## Conclusion

Our findings suggest sex‐divergent relationships between testosterone and fat mass and their distribution but do not support the hypothesis that midlife declines in testosterone lead to lower lean mass. Further prospective observational and experimental studies are required to strengthen causal inferences and investigate the possible adverse cardiometabolic effects of testosterone in women.

## Supporting information

Supplementary Information
