# Supplementary material for: Changes in testosterone related to body composition in late midlife: Findings from the 1946 British birth cohort study
Source: Obesity (Silver Spring). 2015 Jun 5;23(7):1486–92. doi: 10.1002/oby.21092 (PMC4744737; doi:10.1002/oby.21092)
Supplement: Supplementary file 1 — Supplementary Information [file OBY-23-1486-s001.docx]

Supplementary Table 1. Mean differences in body composition outcomes by hormone quartile

Men:

| Hormone | Cut-points  used | N | Fat mass index | Android: gynoid  ratio | Appendicular  lean mass  index + fat mass |
| --- | --- | --- | --- | --- | --- |
| T, 53 | <12.1 | 69 | Referent | Referent | Referent |
| (nmol/l) | 12.1-14.4 | 69 | -0.82(-2.03, 0.39) | 0.97(-4.13, 6.07) | -0.07(-0.35, 0.20) |
|  | >14.4-16.5 | 69 | -1.53(-2.74, -0.33) | -0.88(-5.98, 4.22) | -0.02(-0.30, 0.25) |
|  | >16.5-20 | 69 | -1.48(-2.69, -0.28) | -1.17(-6.27, 3.93) | 0.00(-0.27, 0.28) |
|  | >20 | 69 | -2.02(-3.23, -0.81) | -2.98(-8.08, 2.12) | -0.10(-0.38, 0.18) |
|  |  |  | <0.01 | 0.16 | 0.70 |
| T, 60-64 | <8.8 | 69 |  |  |  |
| (nmol/l) | 8.8-10.9 | 69 | -0.91(-2.05, 0.24) | -2.10(-7.03, 2.83) | 0.07(-0.20, 0.35) |
|  | >10.9-13 | 69 | -1.65(-2.80, -0.51) | -0.98(-5.91, 3.95) | -0.07(-0.35, 0.20) |
|  | >13-15.9 | 69 | -2.85(-4.00, -1.71) | -6.75(-11.69, -1.82) | -0.04(-0.32, 0.25) |
|  | >15.9 | 69 | -3.70(-4.84, -2.55) | -10.77(-15.71, -5.84) | -0.15(-0.44, 0.14) |
|  |  |  | <0.001 | <0.001 | 0.22 |
| SHBG, 53 | <22 | 69 |  |  |  |
| (nmol/l) | 22-28 | 69 | -0.66(-1.88, 0.56) | 0.73(-4.31, 5.78) | 0.04(-0.23, 0.32) |
|  | >28-35 | 69 | 0.08(-1.13, 1.29) | 2.36(-2.64, 7.37) | 0.05(-0.22, 0.32) |
|  | >35-43 | 69 | -1.14(-2.35, 0.08) | -4.83(-9.86, 0.19) | -0.07(-0.34, 0.20) |
|  | >43 | 69 | -1.25(-2.46, -0.03) | -4.51(-9.53, 0.52) | -0.22(-0.49, 0.06) |
|  |  |  | 0.03 | 0.01 | 0.08 |
| SHBG, 60-64 | <24 | 69 |  |  |  |
| (nmol/l) | 24-30 | 69 | 0.05(-1.15, 1.25) | 0.19(-4.74, 5.13) | 0.15(-0.12, 0.41) |
|  | >30-36 | 69 | -1.02(-2.22, 0.18) | -2.82(-7.75, 2.12) | 0.10(-0.17, 0.37) |
|  | >36-45 | 69 | -1.82(-3.02, -0.62) | -6.31(-11.25, -1.38) | -0.24(-0.51, 0.03) |
|  | >45 | 69 | -1.87(-3.06, -0.67) | -10.40(-15.34, -5.47) | -0.22(-0.49, 0.06) |
|  |  |  | <0.001 | <0.001 | 0.01 |
| Free T, 53 | <262 | 69 |  |  |  |
| (pmol/l) | 262-300 | 69 | 0.36(-0.84, 1.55) | 2.07(-2.99, 7.12) | 0.24(-0.03, 0.51) |
|  | >300-345 | 69 | -0.91(-2.10, 0.29) | 0.69(-4.37, 5.74) | 0.11(-0.16, 0.38) |
|  | >342-384 | 69 | -1.99(-3.19, -0.80) | -4.97(-10.02, 0.08) | 0.34(0.06, 0.61) |
|  | >384 | 69 | -1.28(-2.47, -0.08) | 1.02(-4.03, 6.07) | 0.03(-0.24, 0.30) |
|  |  |  | <0.001 | 0.39 | 0.64 |
| Free T, 60-64 | <189 | 69 |  |  |  |
| (pmol/l) | 189-218 | 69 | -1.59(-2.73, -0.44) | -2.52(-7.58, 2.54) | 0.50(0.22, 0.77) |
|  | >218-247 | 69 | -2.09(-3.23, -0.94) | -1.90(-6.97, 3.16) | 0.38(0.10, 0.65) |
|  | >247-291 | 69 | -2.77(-3.91, -1.62) | -3.65(-8.71, 1.41) | 0.24(-0.03, 0.52) |
|  | >291 | 69 | -4.07(-5.21, -2.92) | -6.92(-11.98, -1.86) | 0.21(-0.07, 0.50) |
|  |  |  | <0.001 | 0.01 | 0.65 |

Women:

| Hormone | Cut-points  used | N | Fat mass index | Android: gynoid ratio | Appendicular  lean mass  index + fat mass |
| --- | --- | --- | --- | --- | --- |
| T, 53 | <0.59 | 87 | Referent | Referent | Referent |
| (nmol/l) | >0.59-0.75 | 87 | 2.88(1.35, 4.41) | 5.53(2.01, 9.04) | -0.10(-0.30, 0.09) |
|  | >0.75-0.88 | 87 | 0.73(-0.80, 2.26) | 1.28(-2.24, 4.79) | -0.06(-0.25, 0.14) |
|  | >0.88-1.1 | 87 | 2.31(0.78, 3.84) | 3.24(-0.27, 6.76) | -0.14(-0.34, 0.05) |
|  | >1.1 | 88 | 2.31(0.78, 3.83) | 4.70(1.19, 8.21) | -0.13(-0.33, 0.06) |
|  |  |  | 0.02 | 0.08 | 0.18 |
| T, 60-64 | <0.34 | 87 |  |  |  |
| (nmol/l) | 0.34-0.46 | 87 | 0.65(-0.91, 2.21) | 1.20(-2.37, 4.77) | -0.07(-0.26, 0.12) |
|  | >0.46-0.58 | 87 | 1.10(-0.46, 2.66) | 0.72(-2.85, 4.30) | -0.03(-0.23, 0.16) |
|  | >0.58-0.76 | 87 | 1.43(-0.14, 2.99) | 1.48(-2.10, 5.06) | -0.01(-0.21, 0.18) |
|  | >0.76 | 88 | 0.96(-0.59, 2.52) | 1.27(-2.29, 4.84) | -0.07(-0.26, 0.13) |
|  |  |  | 0.13 | 0.49 | 0.73 |
| SHBG, 53 | <33.5 | 87 |  |  |  |
| (nmol/l) | 33.5-48.6 | 87 | -1.29(-2.79, 0.22) | -5.35(-8.73, -1.97) | -0.12(-0.32, 0.07) |
|  | >48.6-63 | 87 | -3.05(-4.55, -1.55) | -8.35(-11.71, -4.98) | -0.20(-0.39, 0.00) |
|  | >63-91 | 87 | -3.49(-4.99, -1.99) | -10.41(-13.78, -7.04) | -0.16(-0.36, 0.03) |
|  | >91 | 88 | -3.80(-5.30, -2.31) | -10.53(-13.89, -7.17) | -0.29(-0.49, -0.10) |
|  |  |  | <0.001 | <0.001 | <0.01 |
| SHBG, 60-64 | <30.4 | 87 |  |  |  |
| (nmol/l) | 30.4-40 | 87 | -2.81(-4.27, -1.35) | -4.77(-8.00, -1.53) | -0.27(-0.46, -0.07) |
|  | >40-50.6 | 87 | -3.03(-4.49, -1.57) | -6.55(-9.79, -3.32) | -0.18(-0.38, 0.01) |
|  | >50.6-65 | 87 | -3.74(-5.20, -2.28) | -10.59(-13.83, -7.36) | -0.18(-0.37, 0.02) |
|  | >65 | 88 | -5.88(-7.34, -4.43) | -14.76(-17.99, -11.53) | -0.44(-0.64, -0.24) |
|  |  |  | <0.001 | <0.001 | <0.01 |
| Free T, 53 | <6.1 | 87 |  |  |  |
| (pmol/l) | 6.1-8.9 | 87 | 1.61(0.12, 3.10) | 4.37(0.93, 7.81) | 0.00(-0.19, 0.20) |
|  | >8.9-11.6 | 87 | 2.20(0.71, 3.69) | 3.51(0.07, 6.95) | -0.03(-0.23, 0.16) |
|  | >11.6-16.3 | 87 | 2.61(1.12, 4.10) | 6.86(3.42, 10.30) | 0.06(-0.14, 0.25) |
|  | >16.3 | 88 | 4.91(3.42, 6.39) | 9.43(6.00, 12.86) | 0.16(-0.04, 0.37) |
|  |  |  | <0.001 | <0.001 | 0.09 |
| Free T, 60-64 | <4.5 | 87 |  |  |  |
| (pmol/l) | 4.6-6.5 | 87 | 2.16(0.67, 3.65) | 6.26(2.83, 9.68) | 0.10(-0.09, 0.30) |
|  | >6.6-8.3 | 87 | 2.39(0.90, 3.87) | 7.17(3.75, 10.59) | 0.21(0.01, 0.40) |
|  | >8.3-12 | 87 | 4.53(3.04, 6.01) | 7.18(3.76, 10.60) | 0.12(-0.08, 0.32) |
|  | >12 | 88 | 4.13(2.65, 5.62) | 10.35(6.93, 13.76) | 0.20(0.01, 0.40) |
|  |  |  | <0.001 | <0.001 | 0.06 |

Notes: T: testosterone; free testosterone calculated according to Vermeulen et al.

Supplementary Table 2. Mean percentage differences in body mass index (95% CI) at 60–64 years per 1 standard deviation increase in testosterone at 53 and 60-64 years

|  | Men (n=345) |  | P | Women (n=436) | P | P sex  interaction |
| --- | --- | --- | --- | --- | --- | --- |
| Testosterone, 53 (nmol/l) | -2.87(-4.43, -1.32) |  | <0.001 | 2.64(0.94, 4.34) | <0.01 | <0.001 |
| Testosterone, 60-64 (nmol/l) | -4.68(-6.05, -3.30) |  | <0.001 | 1.47(-0.14, 3.09) | 0.07 | <0.001 |
| ∆ Testosterone | -4.37(-5.92, -2.82) |  | <0.001 | 0.43(-1.66, 2.51) | 0.69 | <0.01 |
| ∆ Testosterone, adjusted* | -4.21(-5.79, -2.64) |  | <0.001 | 0.83(-1.28, 2.94) | 0.44 | <0.01 |
|  |  |  |  |  |  |  |
| SHBG, 53 (nmol/l) | -2.63(-4.25, -1.01) |  | <0.01 | -6.10(-7.74, -4.46) | <0.001 | 0.05 |
| SHBG, 60-64 (nmol/l) | -2.83(-4.26, -1.41) |  | <0.001 | -8.47(-10.05, -6.90) | <0.001 | <0.001 |
| ∆ SHBG | -1.77(-3.29, -0.25) |  | 0.02 | -8.04(-10.17, -5.92) | <0.001 | <0.01 |
| ∆ SHBG, adjusted* | -1.54(-3.09, 0.01) |  | 0.05 | -6.98(-9.18, -4.78) | <0.001 | <0.01 |
|  |  |  |  |  |  |  |
| Free testosterone, 53 | -2.83(-4.26, -1.41) |  | <0.001 | 5.95(4.36, 7.53) | <0.001 | <0.001 |
| Free testosterone, 60-64 | -1.75(-3.24, -0.26) |  | 0.02 | 5.14(3.60, 6.68) | <0.001 | <0.001 |
| ∆ Free testosterone | -4.85(-6.71, -2.99) |  | <0.001 | 2.08(0.21, 3.96) | 0.03 | <0.001 |
| ∆ Free testosterone, adjusted* | -4.69(-6.56, -2.81) |  | <0.001 | 2.25(0.41, 4.08) | 0.02 | <0.001 |
|  |  |  |  |  |  |  |

Notes: ∆ standard deviation change between 53 and 60-64 years—analyses adjusted for hormone concentration at 53 years (eg, ∆ testosterone adjusted for testosterone at 53 years); *Adjusted for highest household occupational class, smoking, self-rated health and menopausal status at 53 years; SHBG: sex-hormone binding globulin; free testosterone calculated according to Vermeulen et al; estimated morning testosterone concentrations used at 53 years.

Supplementary Table 3. Mean percentage differences in fat mass (95% CI) at 60–64 years per 1 standard deviation increase in change in testosterone between 53 and 60-64 years, adjusted for hormone concentration at 53 years and body mass index (kg/m^2^) at 53 years

|  | Fat mass index | P | P sex  interaction | Android: gynoid  fat mass ratio | P | P sex  interaction |
| --- | --- | --- | --- | --- | --- | --- |
| Men (N=345) |  |  |  |  |  |  |
| ∆ Testosterone | -6.20(-8.67, -3.72) | <0.01 | 0.02 | -5.16(-7.77, -2.55) | <0.01 | <0.001 |
| ∆ SHBG | -6.32(-8.61, -4.04) | <0.01 | 0.08 | -5.70(-8.11, -3.30) | <0.01 | 0.10 |
| ∆ Free testosterone | -4.17(-7.23, -1.12) | <0.01 | 0.01 | -1.95(-5.16, 1.26) | 0.23 | 0.001 |
|  |  |  |  |  |  |  |
| Women (N=436) |  |  |  |  |  |  |
| ∆ Testosterone | 0.02(-2.20, 2.24) | 0.99 |  | 0.48(-2.43, 3.39) | 0.75 |  |
| ∆ SHBG | -6.87(-9.36, -4.37) | <0.01 |  | -11.19(-14.35, -8.03) | <0.01 |  |
| ∆ Free testosterone | 2.71(0.62, 4.80) | 0.01 |  | 3.77(1.06, 6.47) | <0.01 |  |

Note: free testosterone calculated according to Vermeulen et al

Supplementary Table 4. Mean standard deviation differences in testosterone, sex-hormone binding globulin and free testosterone (95% CI) at 60–64 years per 1 standard deviation increase in change in body mass index between 53 and 60-64 years, adjusted for body mass index at 53 years

|  | Men (n=345) | P | Women (n=436) | P | P sex interaction |
| --- | --- | --- | --- | --- | --- |
|  |  |  |  |  |  |
| Testosterone | -0.17(-0.27, -0.06) | <0.01 | 0.004(-0.11, 0.11) | 0.94 | 0.03 |
| SHBG | -0.21(-0.32, -0.10) | <0.001 | -0.23(-0.33, -0.14) | <0.001 | 0.72 |
| Free testosterone | -0.07(-0.17, 0.04) | 0.22 | 0.10(-0.02, 0.21) | 0.09 | 0.04 |

Note: free testosterone calculated according to Vermeulen et al
